# Response to: Comment on “Antibiotic Resistance Profiles of *Haemophilus influenzae* Isolates from Children in 2016: A Multicenter Study in China”

**DOI:** 10.1155/2021/1541506

**Published:** 2021-02-13

**Authors:** Chun-Zhen Hua, Hong-Jiao Wang, Hui Yu, Chuan-Qing Wang, Ting Zhang, Hong Zhang, Shi-Fu Wang, Ai-Wei Lin, Qing Cao, Wei-Chun Huang, Hui-Ling Deng, San-Cheng Cao, Xue-Jun Chen

**Affiliations:** ^1^Department of Infectious Diseases, The Children's Hospital, Zhejiang University School of Medicine, National Clinical Research Center for Child Health, Hangzhou 310003, China; ^2^Department of Infectious Diseases, Children's Hospital of Fudan University, Shanghai 201102, China; ^3^Department of Clinical Laboratory, Children's Hospital of Fudan University, Shanghai 201102, China; ^4^Department of Infectious Diseases, Children's Hospital of Shanghai Jiaotong University, Shanghai 200240, China; ^5^Department of Clinical Laboratory, Children's Hospital of Shanghai Jiaotong University, Shanghai 200240, China; ^6^Department of Clinical Microbiology, Qilu Children's Hospital of Shandong University, Jinan 250022, China; ^7^Division of Infectious Diseases, Qilu Children's Hospital of Shandong University, Jinan 250022, China; ^8^Division of Infectious Diseases, Shanghai Children's Medical Center of Shanghai Jiaotong University, Shanghai 200127, China; ^9^Department of Clinical Laboratory, Shanghai Children's Medical Center of Shanghai Jiaotong University, Shanghai 200127, China; ^10^Division of Infectious Diseases, Xi'an Children's Hospital, Xi'an 710043, China; ^11^Department of Clinical Laboratory, Xi'an Children's Hospital, Xi'an 710043, China; ^12^Department of Clinical Laboratory, The Children's Hospital, Zhejiang University School of Medicine, Hangzhou 310003, China

We would like to thank Karabay and Karabay [1] for their interest in our study [2].

The first question raised in their letter is whether results obtained from samples collected from sputum and throat swabs would represent profiles of bacteria from infection. The human nasopharynx contains a huge micro ecosystem. A healthy child's nasopharyngeal flora consists of normal flora and conditional pathogenic microorganisms. *Haemophilus influenzae* (*H. influenzae*) is such a conditional pathogen that colonizes the nasopharynx. It is not harmful when it is present in small amounts but is pathogenic when it becomes the dominant colonizer and causes infection. *H. influenzae* is a common cause of community-acquired pneumonia. Culture of lung puncture specimens is the most accurate method for determining the etiology of pneumonia; however, it is rarely used because puncture may lead to trauma. Alveolar lavage fluid culture is another good way for determining the etiology of pneumonia, but again, it is not accepted widely because the procedure for specimen collection requires the use of fiberoptic bronchoscopy examination which is also traumatic. Thus, sputum culture becomes the most commonly used method for etiology diagnosis in pneumonia [3, 4]. This was the reason that we tested *H. influenzae* isolated from sputum culture in our study. As it was isolated from specimens of nonsterile origin, we analyzed whether it was pathological or not before antibiotic susceptibility test was performed. In our study, only those growing in pure culture or as the predominant organism on *Haemophilus* selective plates (with a “+++” or “++++” score obtained from semiquantitative analysis of the streaking inoculation method) were thought to be pathogens [5]. We agree that this definition of pathogens is not as convincing as those from sterile-origin specimens such as blood or cerebrospinal fluid. We also considered this problem and described them as patients with respiratory isolates rather than patients with *H. influenzae* infection in our article. In addition, the objective of our study was to determine the antibiotic resistance profiles of *H. influenzae* isolates from Chinese children and to provide guidelines for clinical treatment. The antibiotic resistance patterns of these pediatric strains would still be useful clinically in providing a reference when treating infection caused by *H. influenzae* even if these strains were a normal colonizer. After all, as *H. influenzae* is a conditional pathogen, the pathogenic bacteria may develop from normal colonizers and their antibiotic resistant patterns would be similar. Furthermore, we have found that the antibiotics-resistant rate of *H. influenzae* from sterile sites was in accordance with that from respiratory tracts isolated in the same year [5, 6].

The second question is what the common causes of vaginitis in children are, and whether *H. influenzae* is the cause for vaginitis in our study. The lower genital tract is another micro ecosystem of the healthy female body, which has a different flora from nasopharyngeal flora. Culture of secretions collected directly from the inflammatory vulva or vagina is useful for the pathogenic diagnosis of vulvovaginitis. A pure growth or heavy growth of a single microorganism species of vulvovaginitis agents in children can be used to diagnose pathogens [7]. In female adults, the most common cause of vaginitis is *Candida*. However, in prepubescent girls, candidal vaginitis is not common because of the neutral vaginal pH which is regulated by sexual hormones and affects the susceptibility to different pathogens. In our study, all patients were from children's hospitals and most patients were prepubescent. Thus, the pathogens of vaginitis or vulvovaginitis were different from those in adult patients. The most common pathogen of pediatric bacterial vulvovaginitis is *Streptococcus pyogenes* [8–10]. *Streptococcus pyogenes* vulvovaginitis in children displays more acute symptoms with painful, erythematous vulva and vagina, mucoid or serosanguineous discharge, and dysuria, and most of the patients may have pharyngitis or tonsillitis, or scarlet fever with raspberry tongue and rash. For *H. influenzae*, it was usually thought to be a respiratory pathogen. However, there have been reports of vulvovaginitis caused by *H. influenzae* [11–13]. We have treated quite a few symptomatic vaginitis patients with heavy growth of single *H. influenzae* strains based on vaginal secretions culture. After treatment with sensitive antibiotics (Oral *β*-lactam antibiotics or topical use of levofloxacin gel), symptoms of vaginitis disappeared and the result of vaginal secretions culture switched to negative. We have also found that *H. influenzae* was isolated more frequently in children with symptomatic vaginitis than healthy children (data not published). We believe that it could be another common cause of pediatric vaginitis, like what has been reported previously [11–13]. *H. influenzae* belongs to fastidious bacteria and could not be cultured successfully without *Haemophilus* selective medium. Therefore, when inoculated vaginal secretions for culture were used, a *Haemophilus* selective plate was advised to be used. *Gardnerella vaginalis* was usually thought to be associated with bacterial vaginosis, rather than vaginitis.
